# Skin grafting while preconditioning with microaxial flow pump: case report of a successful bridge to candidacy approach

**DOI:** 10.1093/ehjcr/ytag137

**Published:** 2026-03-09

**Authors:** Anna Stegmann, Sanas Mirhoseiny, Felix Hennig, Evgenij V Potapov, Pia Lanmüller

**Affiliations:** Department of Cardiothoracic and Vascular Surgery, Deutsches Herzzentrum der Charité, Augustenburger Platz 1, Berlin 13353, Germany; Charité – Universitätsmedizin Berlin, Corporate Member of Freie Universität Berlin and Humboldt-Universität zu Berlin, Charitéplatz 1, Berlin 10117, Germany; Department of Plastic and Reconstructive Surgery, Charité – Universitätsmedizin Berlin, Corporate Member of Freie Universität Berlin and Humboldt-Universität, Charitéplatz 1, Berlin 10117, Germany; Department of Cardiothoracic and Vascular Surgery, Deutsches Herzzentrum der Charité, Augustenburger Platz 1, Berlin 13353, Germany; Charité – Universitätsmedizin Berlin, Corporate Member of Freie Universität Berlin and Humboldt-Universität zu Berlin, Charitéplatz 1, Berlin 10117, Germany; German Center for Cardiovascular Research (DZHK), Partner site Berlin, Potsdamer Str. 58, Berlin 10785, Germany; Department of Cardiothoracic and Vascular Surgery, Deutsches Herzzentrum der Charité, Augustenburger Platz 1, Berlin 13353, Germany; Charité – Universitätsmedizin Berlin, Corporate Member of Freie Universität Berlin and Humboldt-Universität zu Berlin, Charitéplatz 1, Berlin 10117, Germany; German Center for Cardiovascular Research (DZHK), Partner site Berlin, Potsdamer Str. 58, Berlin 10785, Germany; Department of Cardiothoracic and Vascular Surgery, Deutsches Herzzentrum der Charité, Augustenburger Platz 1, Berlin 13353, Germany; Charité – Universitätsmedizin Berlin, Corporate Member of Freie Universität Berlin and Humboldt-Universität zu Berlin, Charitéplatz 1, Berlin 10117, Germany; German Center for Cardiovascular Research (DZHK), Partner site Berlin, Potsdamer Str. 58, Berlin 10785, Germany

**Keywords:** Heart failure, Microaxial Flow Pump, Left Ventricular Assist Device, Case Report, Bridge to Candidacy, Heart transplantation, Skin grafting

## Abstract

**Background:**

Microaxial flow pumps (mAFP) are a well-established treatment modality for patients in cardiogenic shock, significantly improving haemodynamic and end-organ-function. In complex heart failure patients currently ineligible for advanced heart failure therapies, such as durable left ventricular assist device (dLVAD) implantation or heart transplantation (HTx), mAFP can serve as a preconditioning tool in a bridge-to-candidacy strategy.

**Case summary:**

A 61-year-old male patient with known dilatative cardiomyopathy and multiple comorbidities presented in cardiogenic shock, accompanied by end-organ dysfunction, fluid overload, and the need for moderate catecholaminergic support. Bilateral lower limb stasis dermatitis with infected ulcers excluded advanced heart failure surgery. Initial management included high-dose diuretics, paracenteses and pleural drainage. To further improve microcirculation and optimize conditions for ulcer treatment, ongoing haemodynamic support was escalated through the implantation of a full-support transaxillary mAFP. Comprehensive wound care was initiated with successful split-thickness skin grafting during ongoing mAFP support. Given favourable wound healing, the patient underwent uncomplicated dLVAD implantation. Early postoperative right heart failure necessitated continued inotropic support. Following high-urgency HTx listing the patient was successfully transplanted eight months later.

**Conclusion:**

Medium-term full-support mAFP provided robust circulatory output, enabling effective decongestion and wound healing in a bridge-to-candidacy approach.

Learning points

- Patients with advanced heart failure who are ineligible for LVAD implantation or heart transplantation due to reversible contraindications may benefit from complex preconditioning strategies.
- Microaxial flow pumps provide robust haemodynamic support for interventions such as skin grafting and optimize conditions for effective wound healing in a bridge-to-candidacy approach.
- Early Right heart failure after dLVAD implantation may occur despite stable right heart function under mAFP support.

## Introduction

Temporary mechanical circulatory support devices such as micro-axial flow pumps (mAFP) have proven effective in bridging patients with end-stage heart failure to definitive therapy.^1,2^ In the setting of cardiogenic shock (CS), the use of mAFP is associated with lower in-hospital mortality, stroke incidence, and device-related complications compared with venoarterial extracorporal membrane oxygenation (VA-ECMO) and allows optimization of end-organ function and volume status prior to advanced interventions such as heart transplantation (HTx) or durable left ventricular assist device (dLVAD) implantation.^3–6^ In patients who are not immediately eligible for HTx or dLVAD implantation due to reversible contraindications—such as ongoing infections, malnutrition, psychosocial factors, impaired secondary organ function or reversible right heart failure—mAFP-supported preconditioning may represent the only viable pathway toward candidacy.^4,7^

We present the case of a patient with advanced chronic heart failure and infected low extremity ulcers, successfully managed during prolonged mAFP support with subsequent bridge-to-candidacy for dLVAD and ultimately HTx.

## Summary figure

**Figure:**
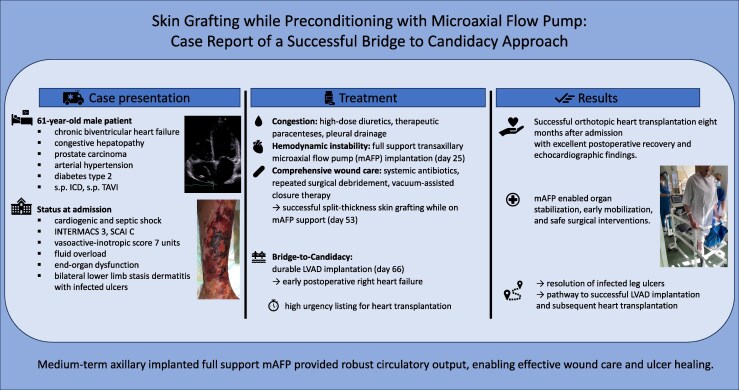


## Case presentation

A 61-year-old male patient (186 cm, 98.1 kg, blood group B) was transferred to our tertiary care cardiac centre in CS secondary to chronic biventricular heart failure. His comorbidities included arterial hypertension, type 2 diabetes mellitus, dyslipidemia and prostate carcinoma (Gleason score 7, cT1c, non-metastatic), which had been diagnosed 5 years earlier and was recently treated with curative intent using radiation. The patient was already under outpatient follow-up at our centre for dilatative cardiomyopathy with recurrent decompensation with ascites and pleural effusions. Prior treatment approaches included PCI for single-vessel coronary artery disease (6 years prior), prophylactic CRT-D implantation for LBBB (5 years prior), and TAVI for low-flow, low-gradient aortic valve stenosis (2 years prior). Htx had previously been discussed with the patient, who declined listing at the time, citing a perceived good quality of life.

**Figure 1:**
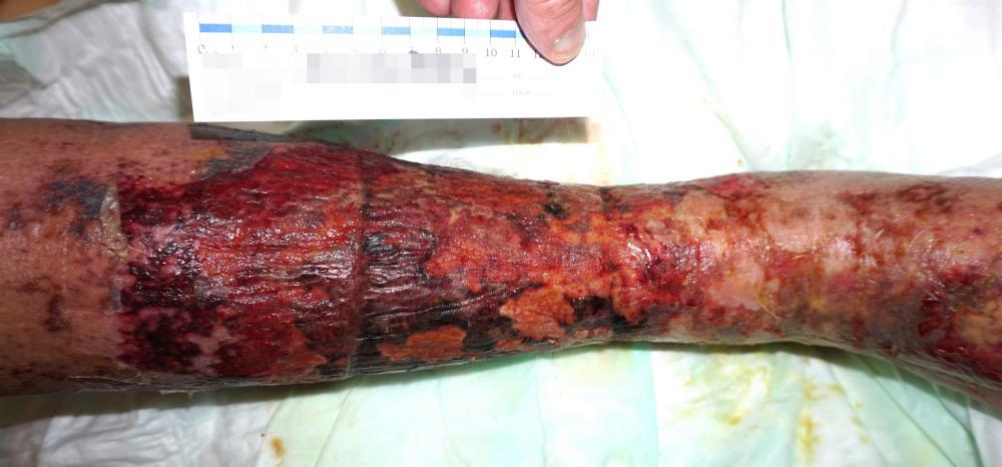
Bilateral lower leg hemosiderosis and right-sided ulceration at presentation.

On the recent admission, he required moderate catecholamine support for hypotension [INTERMACS level 3, SCAI stage C, vasoactive-inotropic score (VIS) 7 units]. NT-proBNP was elevated to 19 868 pg/mL. Bilateral lower leg stasis dermatitis had progressed to right-sided infected ulcers (*Escherichia coli*, *Figure 1*). As signs of systemic infection, inflammatory markers were markedly elevated (C-reactive protein 14.8 mg/dL, procalcitonin 2.6 ng/mL) and a septic component was considered. End-organ dysfunction was evident with acute kidney injury (creatinine 3.2 mg/dL) and congestive hepatopathy (GGT 254.2 U/L, cholinesterase 1597 U/L, total bilirubin 3.6 mg/dL). Transthoracic echocardiography revealed a severely dilated left ventricle (LVEDD 67 mm), poor left ventricular ejection fraction (LVEF 28%), moderately impaired right ventricular fraction (CVP 21 mmHg), moderate tricuspid regurgitation, and mild mitral regurgitation (see Supplement  *S1*).

Initial decongestion with diuretics and repeated paracenteses led to a 16 kilograms weight reduction over 4 weeks, resulting in normalization of central venous pressure (CVP) and improvement in liver function. Inflammatory markers decreased under systemic targeted antibiotic therapy; however a plastic surgery consultation for the lower leg ulcers deemed the wounds unsuitable for reconstruction due to extensive necrotic tissue.

**Figure 2:**
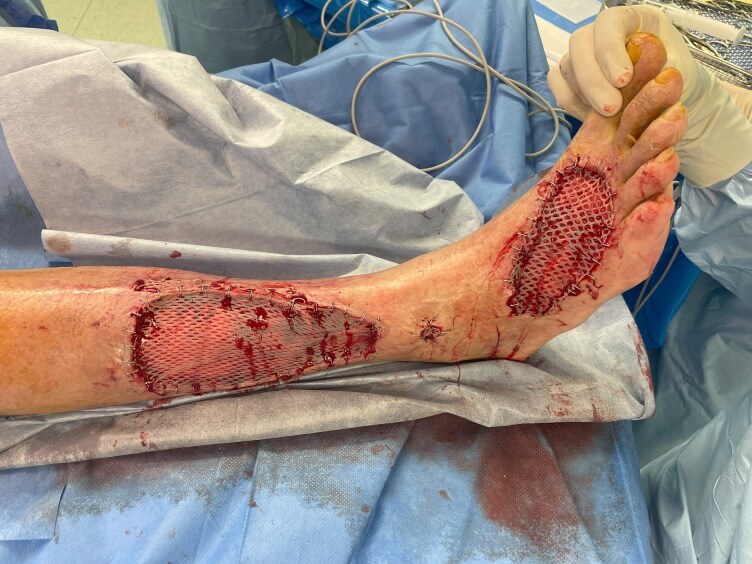
Protected split-thickness skin grafting of the right lower leg and foot under mAFP support.

With the goal of further preconditioning, especially targeting microcirculatory dysfunction in the presence of skin defects, the institutional heart failure board decided for a treatment approach with additional mAFP implantation, as the patient remained catecholamine-dependent (VIS 5, ZVS 56%). An Impella 5.5 (Abiomed, USA) was implanted via the right axillary artery under local anaesthesia on the day 25th day of intermediate care (IMC) stay. Following implantation, the plastic surgery and wound care teams initiated a treatment strategy, including repeated surgical debridement and vacuum-assisted closure (VAC) therapy. The risk of deterioration of the cutaneous condition under anticoagulation (target aPTT 55–65 s) was carefully evaluated. As local necrosis debridement improved wound conditions, the patient was subsequently approved for skin grafting by the plastic surgery team. On hospital day 53, ∼1 month after mAFP implantation, split-thickness skin grafting was successfully performed on the right lower leg and foot (*Figure 2*, Supplement  *S2*). Bleeding complications occurred and although they did not delay grafting or wound healing, they required 3 additional superficial wound revisions under VAC therapy and 7 units of red blood cell transfusions over a 23-day period of advanced wound care.

**Figure 3:**
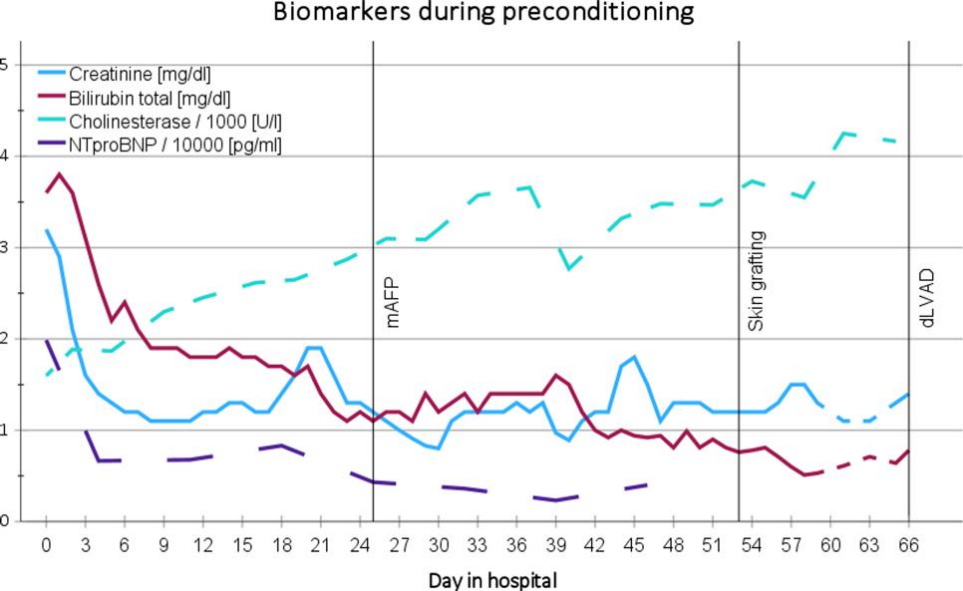
Trends in cardiac, renal and hepatic biomarkers during mAFP support.

Preconditioning included a nutritional rehabilitation programme and early mobilization under mAFP support, facilitated by physiotherapists and cardiac rehab teams (see Supplementary material online, *Video S1*). End-organ function improved steadily (*Figure 3*). With favourable wound healing (see Supplement  *S2*) and stable right ventricular function (RVEF 41%) under mAFP on IMC day 66, 6 weeks after mAFP insertion, the patient underwent dLVAD implantation. Intraoperatively, the dLVAD speed was set to 5200 RPM under transoesophageal echocardiographic guidance demonstrating an optimally decompressed right ventricle. This speed resulted in dLVAD flows between 4.4 and 5.0 L/min with adequate left ventricular unloading (LVEDD 49 mm, PCWP 9 mmHg). Initial postoperative recovery was favourable and assisted full mobilization was achieved by day 8. Despite the intraoperative adequately unloaded right ventricle, echocardiography follow-up 5 days after implantation revealed progressive right ventricular dilatation and recurrent volume overload, necessitating ongoing inotropic therapy. High-urgency listing for HTx became necessary due to persistent right heart failure on LVAD. Right heart catheterization revealed no signs of pulmonary hypertension [PAP 32/14/20 mmHg; PCWP 10 mmHg; PVR 1.6 WU; CVP 15 mmHg; CI (FICK) = 1.89 L/min/m^2^]. Meanwhile, the patient’s previously treated prostate carcinoma was further managed with hormone therapy and osteoprophylaxis. Eight months post-LVAD, he underwent successful orthotopic bicaval heart transplantation and was discharged to rehabilitation with excellent echocardiographic findings (see Supplement  *S2*).

## Discussion

Currently, the only available long-term therapies for advanced heart failure are the implantation of a durable LVAD or heart transplantation.^2^ To reduce complications and improve outcomes, careful patient selection is essential, as systemic infections and untreated malignancies may serve as contraindications.^4,7^

Stasis dermatitis and venous ulcers are prevalent among patients suffering from longstanding biventricular heart failure and are associated with inflammatory and microvascular dysfunction.^8,9^ Infection of these ulcers, as in this case, often preclude immediate surgical intervention.

The mAFP allowed safe circulatory support via minimally invasive axillary access implanted under local anaesthesia, enabling early mobilization and nutritional optimization. Termination of inotropic therapy and improved cardiac output enhanced decongestion and subpapillary skin perfusion, thereby promoting ulcer healing and enabling skin grafting.^10^ Following ulcer healing, the patient became eligible for LVAD implantation. In patients with biventricular heart failure, preconditioning with mAFP, resulting in right ventricular improvement under left ventricular unloading, can enable long-term stable conditions under LVAD support in selected cases.^11^ In the present case, preconditioning with mAFP resulted in improved end-organ function and stable right ventricular performance; consequently, the institutional heart failure board opted for isolated dLVAD implantation. Although adequate right ventricular unloading was confirmed by intraoperative echocardiography under dLVAD support, subsequently the patient developed early right heart failure and was listed for HTx with high-urgency status. Following treatment and in accordance with updated guidelines, prostate carcinoma was no longer considered a contraindication.^12^ Despite prolonged mechanical support, no donor-specific antibodies were detected, and transplant outcomes were favourable.

## Conclusion

Microaxial flow pumps are a valuable tool in the bridge-to-candidacy approach for advanced heart failure therapies for patients with reversible contraindications. They enable organ stabilization, early mobilization, and safe surgical interventions. In this case, mAFP facilitated the resolution of infected leg ulcers and created a pathway to successful LVAD implantation and subsequent heart transplantation.

## Supplementary Material

ytag137_Supplementary_Data

## Data Availability

All relevant data are within the manuscript and its Supporting Information files.

## Lead author biography

Anna Stegmann joined the Department of Cardiothoracic and Vascular Surgery at Deutsches Herzzentrum der Charité (DHZC) in 2021, where she has since contributed to both clinical care and academic research in the field of advanced heart failure and mechanical circulatory support. Her work focuses on complex surgical cases involving left ventricular assist devices (LVADs), heart transplantation, and temporary support strategies. In collaboration with a multidisciplinary team, she has been involved in innovative treatment approaches for critically ill patients.
