# Supplementary material for: Skin grafting while preconditioning with microaxial flow pump: case report of a successful bridge to candidacy approach
Source: Eur Heart J Case Rep. 2026 Mar 9;10(3):ytag137. doi: 10.1093/ehjcr/ytag137 (PMC13007766; doi:10.1093/ehjcr/ytag137)
Supplement: ytag137_Supplementary_Data [file ytag137_supplementary_data.zip › Supplement_1.pdf]

Supplement 1: Timeline of echocardiographic findings.

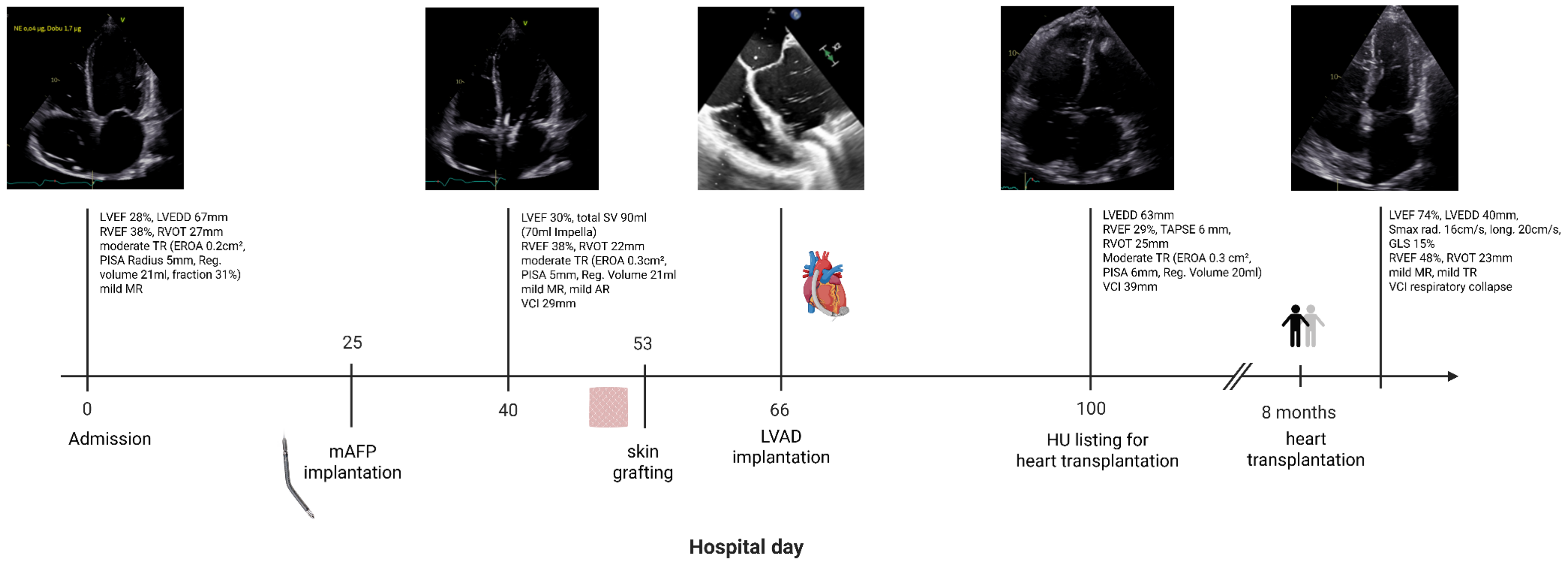

Abbreviations:

AR: Aortic Regurgitation. **EROA**: Effective Regurgitant Orifice Area. **GLS**: Global Longitudinal Strain. **IMC**: Intermediate Care Unit. **LVAD**: Left Ventricular Assist Device. **LVEDD**: Left Ventricular End-Diastolic Diameter. **LVEF**: Left Ventricular Ejection Fraction. **mAFP:** Microaxial Flow Pump. **MR**: Mitral Regurgitation. **PISA**: Proximal Isovelocity Surface Area. **Reg. Fraction**: Regurgitant Fraction. **Reg. Volume**: Regurgitant Volume. **RVOT**: Right Ventricular Outflow Tract diameter. **RVEF**: Right Ventricular Ejection Fraction. **Smax long.**: Maximum Longitudinal Systolic Velocity. **Smax rad.**: Maximum Radial Systolic Velocity. **SV**: Stroke Volume. **TAPSE**: Tricuspid Annular Plane Systolic Excursion. **TEE**: Transesophageal Echocardiography. **TR**: Tricuspid Regurgitation. **TTE**: Transthoracic Echocardiography. **VCI**: Vena Cava Inferior.

Created in BioRender.
