# Supplementary figures and images for: Skin grafting while preconditioning with microaxial flow pump: case report of a successful bridge to candidacy approach
Source: Eur Heart J Case Rep. 2026 Mar 9;10(3):ytag137. doi: 10.1093/ehjcr/ytag137 (PMC13007766; doi:10.1093/ehjcr/ytag137)

**Supplement 2:** Wound documentation of leg ulcers before and after skin transplantation.

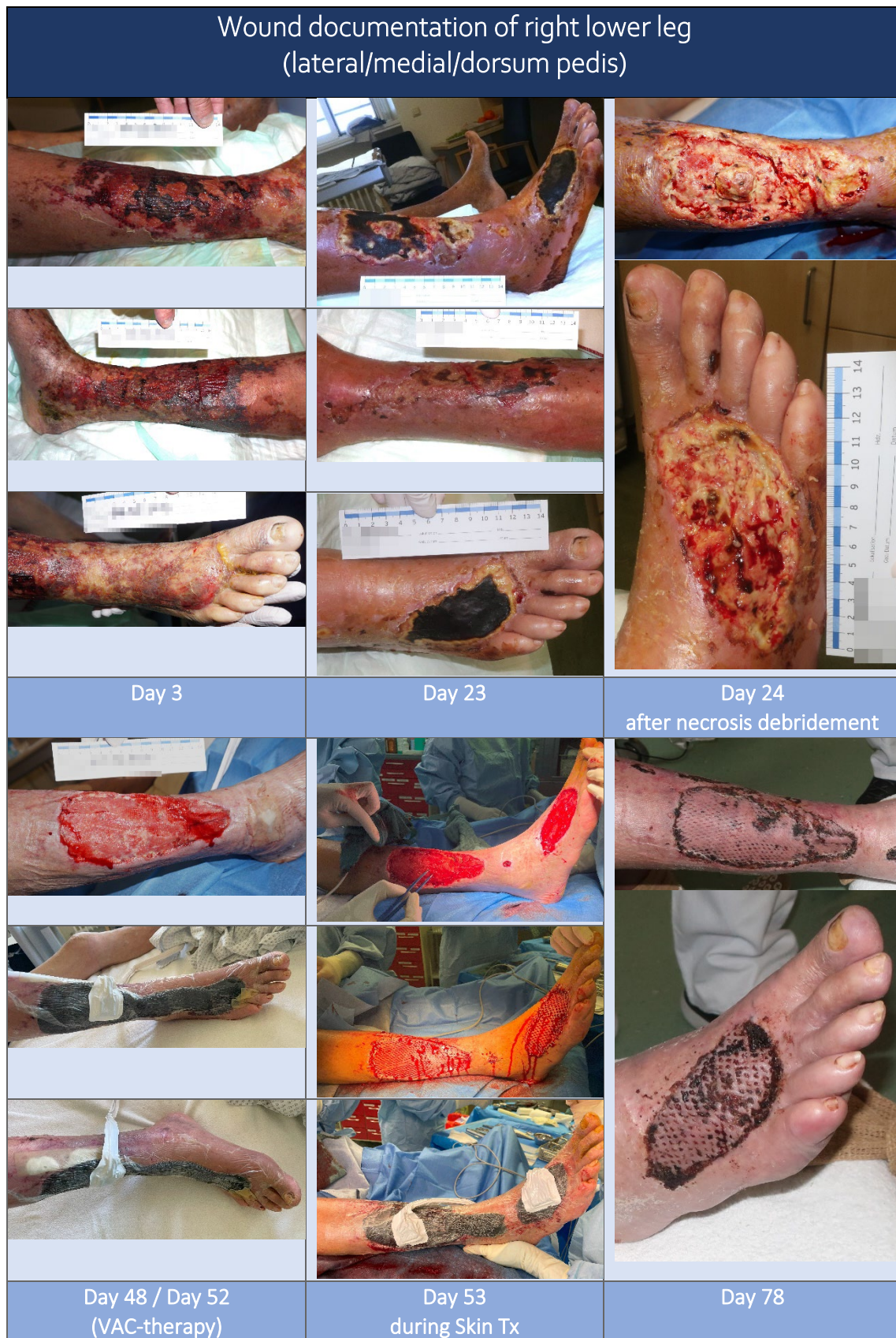

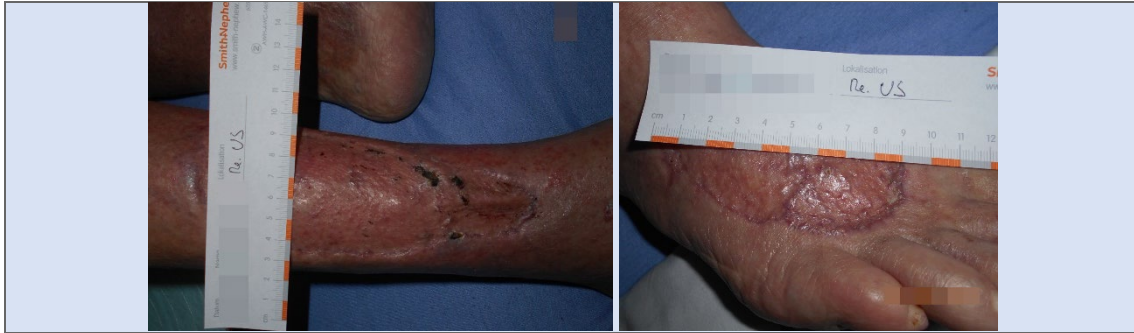

Day 118

Supplement: ytag137_Supplementary_Data [file ytag137_supplementary_data.zip › Supplement_2.pdf]
